# Comparative Transcriptome Analysis Revealing the Potential Mechanism of Low-Temperature Stress in *Machilus microcarpa*

**DOI:** 10.3389/fpls.2022.900870

**Published:** 2022-07-19

**Authors:** Xinru He, Fengying Long, Yingjie Li, Yaowen Xu, Longsheng Hu, Tianshu Yao, Yingying Huang, Die Hu, Yujie Yang, Yongjun Fei

**Affiliations:** College of Horticulture and Gardening, Yangtze University, Jingzhou, China

**Keywords:** *Machilus microcarpa*, cold tolerance, transcriptome, RNA-Seq, differentially expressed genes

## Abstract

*Machilus microcarpa* is a rare national tree species in China and possesses important ornamental and ecological value. *M. microcarpa* can be planted in low-temperature areas, depending on whether its seedlings can withstand the harm. To face this problem, the annual seedlings of *M. microcarpa* were subjected to five temperature treatments, and eight physiological indicators were measured. Furthermore, comparative transcriptome analysis was performed between *M. microcarpa* leaves treated at 25°C and −2.8°C. A total of 9,385 differentially expressed genes (DEGs) were involved in low-temperature stress in *M. microcarpa*. An upregulated (*cobA*) and five downregulated (*HEM, CHLM, CRD, CLH*, and *PORA*) genes associated with the porphyrin and chlorophyll metabolism pathway may reduce chlorophyll synthesis under low-temperature stress. Upregulation of six DEGs (two *GAPDHs, PFK, PGAM, PDC*, and *PK*) involved in the glycolysis/gluconeogenesis pathway provided energy for *M. microcarpa* under adverse cold conditions. Thirteen upregulated and seven downregulated genes related to antioxidant enzymes were also observed under low-temperature stress. Candidate transcription factors (TFs) played key roles in signal transduction under low-temperature stress in *M. microcarpa*, and quantitative real-time PCR (qRT-PCR) analysis validated the RNA-seq data. The results provide valuable information for further studies on the cold response mechanisms for low-temperature stress in *M. microcarpa*.

## Introduction

*Machilus microcarpa* is a woody plant of the Lauraceae family. It is officially listed as a national rare tree species in China, where it is extensively distributed in the Sichuan, Hubei, and Guizhou provinces. It is an excellent wood species with many ecological functions, such as dust retention, insect repellent, and noise reduction. It is evergreen all year round, and its new leaves are bright red; its seeds reproduce easily and grow rapidly. It can be cultivated as an urban street tree and is a landscaping tree species with great development potential. In recent years, studies have focused on the leaf epidermis structure, dust-retention ability (He et al., 2019), structure characteristics (Chen et al., 2015), and transpiration characteristics (Li et al., 2019a) of *M. microcarpa*.

For a long time, some excellent garden trees in the south have been introduced and cultivated from the south to the north at high latitudes. Due to temperature restrictions, they cannot adapt to the local climate and have difficulty overwintering. Exploring the cold resistance mechanism of plants is the basic work of plant breeding, reasonable introduction, and cold-resistant cultivation. It is one of the important components of botany research and has important economic value for reducing natural disasters. Different *Machilus* plants show different adaptabilities to low-temperature stress. A previous research has shown that *M. microcarpa* has strong cold tolerance in Hubei Province, which is an ideal material for studying the low-temperature tolerance of *Machilus*. Therefore, elucidating the response mechanism of this species to low-temperature stress is essential for future breeding concerning the cold tolerance of *M. microcarpa*. Among the various abiotic stresses, low temperature is one of the most common environmental stresses that seriously affect the growth and development of plants (Megha et al., 2018). Under low temperatures, plants will perform a series of adaptive and self-protection processes, such as changes in membrane permeability, accumulation of osmolytes, and an increase of antioxidants (Zhang et al., 2016). Malonaldehyde is the final product of lipid peroxidation, and its content shows the extent of damage to the membrane (Rakei et al., 2016). Soluble sugars and soluble proteins accumulated in plants serve as cryoprotectants, which can decrease the freezing point of water in seedlings (Meng et al., 2015; Farhangi-Abriz and Torabian, 2017). The activity of superoxide dismutase, peroxidase, and polyphenol oxidase can remove redundant reactive oxygen species (ROS) and detoxify their harmful effects (Gill and Tuteja, 2010). The soluble protein content and enzymatic activity increase under low-temperature stress, regulating the plant's cold hardiness, according to Chen et al. (2014).

The RNA-seq technology plays an important role in the research progress of plants' cold resistance. By analyzing the transcriptome of plant samples under low-temperature stress, DEGs can be identified and isolated to identify cold resistance genes. Under low-temperature stress, genes can regulate many functions of plants and make the expression of related genes resist stress (Liu et al., 2013; Baxter et al., 2014). Currently, RNA-seq has been widely used in *Eucalyptus nitens* (Gaete-Loyola et al., 2017), *Arabidopsis thaliana* (Fowler and Thomashow, 2002), *Olea europaea* (Guerra et al., 2015), and *Triticum aestivum* (Laudencia-Chingcuanco et al., 2011; Xiong et al., 2017) in low-temperature. To clarify the responses of the expansin gene in a highly cold-tolerant winter wheat variety (D2) to low-temperature stress, the transcriptome of plants treated at 4°C was analyzed by Feng et al. (2019), which showed that the expression level of *TaEXPB7-B* responded to low-temperature stress. In addition, *TaEXPB7-B* enhanced antioxidants and osmotic regulation in transgenic *Arabidopsis*, thus, improving the tolerance and survival rates of plants under low-temperature stress.

In this study, the physiological indexes under different temperature treatments were measured for *M. microcarpa*. Through RNA-seq technology, which was used to conduct a comprehensive analysis of transcriptional responses, cold response genes were identified, and the regulatory mechanism of low temperature in *M. microcarpa* was analyzed, thus, having great economic value for reducing the natural disasters of *M. microcarpa* under low temperature. New gene resource for genetic improvement was provided, and the molecular mechanism of cold tolerance formation was clarified, accumulating biological knowledge of *M. microcarpa*.

## Materials and Methods

### Plant Materials and Treatments

In this study, annual *M. microcarpa* plants were grown at the Germplasm Resources Evaluation and Innovation Center of *Phoebe*, Yangtze University, Jingzhou, China. A total of 450 independent plants with uniform growth and no pests or diseases were collected. There were three biological replicates of 30 plants each. Annual *M. microcarpa* plants in the artificial intelligence incubator were cooled at a rate of 1°C/d to reach different target temperatures (2, 1, 5, 0, and −5°C). Plants were kept at each temperature for 24 h. Then, the leaves were removed from the same part of the plant and stored at −80°C in an ultra-low temperature freezer (Haier, China) until further use.

### Determination of Growth and Physiology Characteristics

The measurement of relative electric conductivity (REC) followed Feng et al. (2005). The temperature at 50% REC was defined as the lethal temperature of the tissues (LT_50_). The total chlorophyll content was determined as described by Wellburn (1994). The malondialdehyde (MDA) content was determined using the thiobarbituric acid (TBA) method (Li et al., 2010). Soluble sugar content was measured using the anthrone colorimetric method (Moustakas et al., 2011). Total soluble protein content was determined using the Bradford method (Bradford, 1976). The activities of antioxidant enzymes, including superoxide dismutase (SOD), peroxidase (POD), and polyphenol oxidase (PPO) were determined using kits (Nanjing Jiancheng Bioengineering Institute, China).

### RNA Extraction and Transcriptome Sequencing

The tender leaves were removed after being treated at 25°C and −2.8°C (LT_50_) for 24 hours, and three biological replicates were set. The sample treated at 25°C was called C, and the sample treated at −2.8°C was called M. Six RNA samples (C-1, C-2, C-3, M-1, M-2, and M-3) were extracted using the EASY spin Plus Plant RNA Kit (AidLab, China) according to the protocol. RNA sample purity was measured using a NanoPhotometer spectrophotometer, and the integrity and RNA sample integrity and concentration were checked using an Agilent 2100 RNA Bioanalyzer. The concentration and quality of these libraries were evaluated on the Agilent 2100 bioanalyzer and the Qubit2.0 fluorometer. All the samples were sequenced on the Illumina HiSeq X-ten platform, which was performed by Beijing Nuohe Zhiyuan Biotechnology Co., Ltd. Raw image data from Illumina HiSeq X-ten was transformed to raw reads by CASAVA base recognition and stored in FASTQ format. To obtain high-quality clean data, the raw reads were filtered, mainly to remove reads with sequencing adapters, reads containing indeterminate base information, and low-quality reads (Q_phred_ ≤ 20 for >50% read).

### Functional Gene Annotation

A transcriptome assembly for all clean reads was done using Trinity (Grabherr et al., 2011) with min-kmer-cov set to 2 by default and all other parameters set at default (Liu et al., 2022). The functional annotation of transcriptome assembly sequences was annotated based on the following databases: Non-redundant (Nr), Nucleotide sequences (Nt), Protein sequence (Swiss-Prot), Kyoto Encyclopedia of Genes and Genomes (KEGG), Clusters of Orthologous Groups of proteins (KOG), and Gene Ontology (GO).

### Differentially Expressed Gene Analysis

The assembled transcriptome was used as a reference database, and all clean reads were mapped back to the reference transcriptome by Bowtie 2, the read count for each gene was derived from the mapping results using RSEM (Nan et al., 2018). Following that, the fragments per kilobase of an exon in per million fragments mapped reads (FPKM) values were used to represent the expression abundance of the reading frame corresponding to the unigenes (Song et al., 2019). In this study, we used DESeq to compare the treatment group with the control group and selected a false discovery rate (FDR) of <0.01 and a fold change (FC) of ≥ 2, which indicates differential expression (Gou et al., 2020). The DEGs were screened by pairwise comparison of the two libraries, C and M, for gene function enrichment analysis and metabolic pathway enrichment analysis.

### qRT-PCR Analysis

Total RNA isolation was extracted using an EASY spin Plus Plant RNA Kit (RN38, AidLab, China). First-strand cDNA was synthesized with HiScript II Q RT SuperMix for qPCR (+gDNA wiper) (R223, Vazyme, Nanjing, China) and the extracted RNA was used as the template. The Ef-1a-C11786.0 was selected as the reference gene of M. microcarpa, and primers were designed using Primer 6.0 (Supplementary Table S1). qRT-PCR was performed using the LineGene 9600 Plus Fluorescent Quantitative PCR System (Bioer, Hangzhou, China) and the ChamQ SYBR qPCR Master Mix (Without ROX, Q311, Vazyme). Each sample was performed on three biological replicates and three technical replicates to ensure reproducibility and reliability. The reaction system was 20 μl: 10 μl of 2 × ChamQ SYBR qPCR Master Mix, 1 μl of sense primer (10 μM), 1 μl of antisense primer (10 μM), 2 μl of cDNA, and 6 μl of RNase-free water. The PCR program was as follows: 95°C for 30 s, 40 cycles at 95°C for 10 s, and 60°C for 30 s, followed by 95°C for 15 s, 60°C for 60 s, and 95°C for 15 s. The relative expression values were calculated using the 2^−Δ*ΔCt*^ method (Schmittgen and Livak, 2008).

### Statistical Analysis

The treatment temperature and REC were fitted with the logistic function to obtain the inflection point temperature of the curve to determine the LT_50_ of tissue (Li et al., 2017; Wang et al., 2018). The logistic regression function was Y = K/(1 + ae ^−*bx*^), LT_50_ = lna / b, where Y represents REC at low temperature; x is the treatment temperature; and K, a, and b are the function parameters. K was set to 100, indicating the saturation value of Y. The corresponding relationship between x and Y is represented by b.

Data were analyzed in Microsoft Excel 2019 and using analysis of variance (ANOVA) in SPSS 22.0, followed by Duncan's significant difference test at *p* ≤ 0.05. Each dataset had three biological replicates.

## Results

### Effects of Different Temperature Treatments on Physiological Changes in *M. microcarpa* Leaves

**Figure 1:**
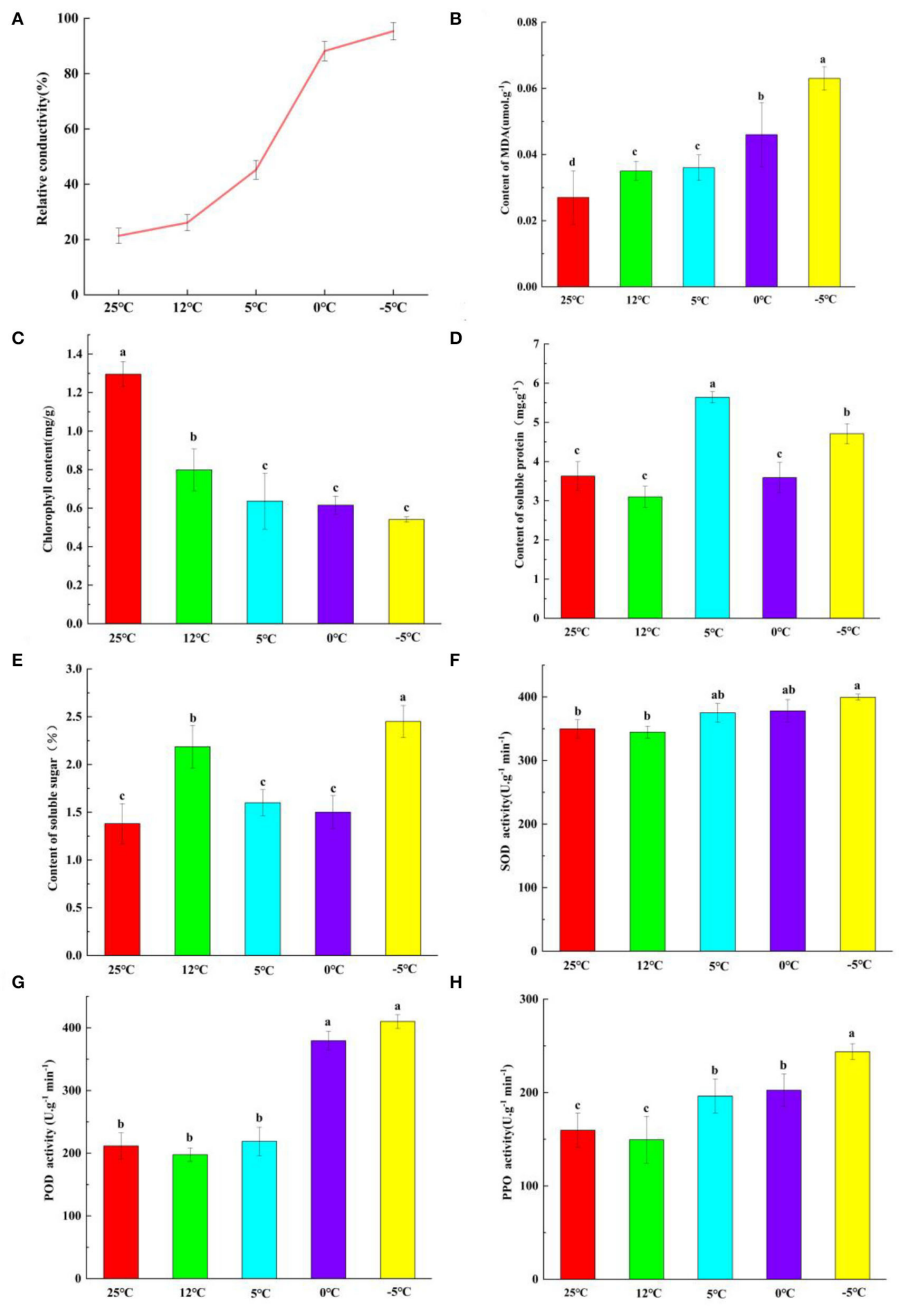
Comparison of physiological indexes of *M. microcarpa* leaves treated by five temperature treatments. **(A–H)** The relative electric conductivity, MDA content, chlorophyll content, soluble protein content, soluble sugar content, SOD, POD, and PPO activities were compared under five temperature treatments of *M. microcarpa* leaves, respectively. All data were shown as mean ± SE (*n* = 3). Within each treatment, means with different letters were significantly different at *p* < 0.05.

The REC of *M. microcarpa* leaves increased with a decrease in temperature (Figure 1A). At 25°C, the REC was the lowest at 21.37%. At 0°C, the REC rapidly increased to 88.16%. At −5°C, the REC was more than 90%. The data on the logistic functions and LT_50_ showed that the function's fitting degree was 0.92, reaching an extremely significant level. The linear equation was y = 100/(1 + 1.15e^0.05x^), and the LT_50_ of *M. microcarpa* was attained at −2.8°C.

Malondialdehyde (MDA) is a product of cell membrane peroxidation, and its content determines the degree of damage to the cell membrane. The MDA content of *M. microcarpa* leaves increased with a decrease in temperature (Figure 1B). At 25°C, the MDA content was the lowest. At −5°C, the MDA content was the highest, and there were significant differences from the other treatment groups.

In *M. microcarpa* leaves, the total chlorophyll content decreased with decreasing temperature (Figure 1C), and the contents of soluble proteins (Figure 1D) and soluble sugars (Figure 1E) changed irregularly. At 25°C, the total chlorophyll content was the highest, and the soluble sugar content was the lowest. At −5°C, the total chlorophyll content was the lowest, and the soluble sugar content was the highest. The soluble protein content was the lowest at 12°C. The soluble protein content was the highest at 5°C, and it was significantly different from the other groups. With decreasing temperature, the changes in SOD (Figure 1F), POD (Figure 1G), and PPO (Figure 1H) activities were consistent in the *M. microcarpa* leaves. The POD, SOD, and PPO activities were the lowest at 12°C. In contrast, when the treatment temperature was −5°C, the POD, SOD, and PPO activities were the highest.

### Correlation Analysis of Physiological Indexes Under Different Temperature Treatments in *M. microcarpa*

To further investigate the relationships among these physiological indicators, a correlation analysis was performed (Supplementary Table S2). POD activity was positively correlated with soluble protein, and the correlation coefficient was.596. SOD activity was positively correlated with soluble protein and POD activity (0.583 and 0.524, respectively). PPO activity was positively correlated with soluble protein, had a highly significant positive correlation with POD and SOD activities, and had a highly significant negative correlation with chlorophyll content (0.595, 0.758, 0.686, and −0.684, respectively). Soluble sugar had a highly significant negative correlation with PPO activity and a positive correlation with chlorophyll content; the correlation coefficients were 0.786 and −0.692, respectively. POD, SOD, and PPO activities and soluble sugar all played an important role in the removal of ROS in *M. microcarpa* leaves, and reached a significant or highly significant level, indicating that antioxidants play a more important role in the removal of ROS in the *M. microcarpa* leaves under low-temperature treatments.

### Recovery of *M. microcarpa* After Treatment at Different Temperatures

**Figure 2:**
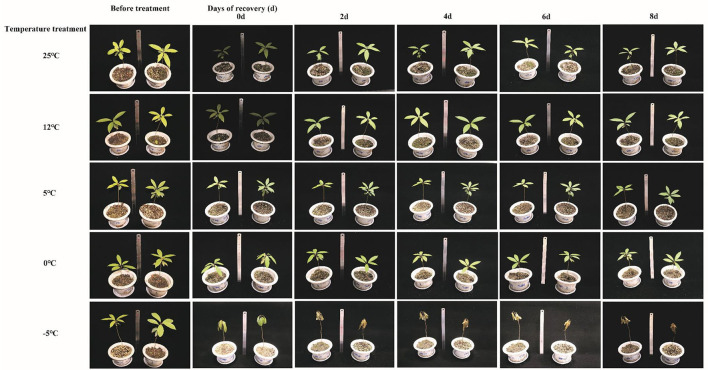
Recovery of *M. microcarpa* after being treated at different temperatures.

As shown in Figure 2, when treated at 25, 12, and 5°C, *M. microcarpa* leaves showed no significant change before and after temperature treatment. When *M. microcarpa* was treated at 0°C and recovered for 0 days, the leaves showed slight drooping. However, after 2 days of recovery, *M. microcarpa* leaves returned to normal, and there was no significant change from before treatment. When *M. microcarpa* was treated at −5°C and recovered for 0 days, the leaves underwent intercellular freezing. After 2 days of recovery, the leaves and stem segments dried up, and the plant died.

### Overview of the Transcriptome Data

In this study, a total of 60.50 Gb clean data were obtained by analyzing the leaves of *M. microcarpa*. Each sample produced over 9.64 Gb of clean data. The Q30 percentage was over 93.10%, and the GC content was in the range of 46.14–47.16%. After filtering out low-quality reads, an average of 67,233,156 reads were obtained for each sample (Supplementary Table S3). The results showed that the sampling of *M. microcarpa* was reliable and suitable for further analysis in this study.

### Functional Annotation and Classification of *M. microcarpa* Unigenes

**Figure 3:**
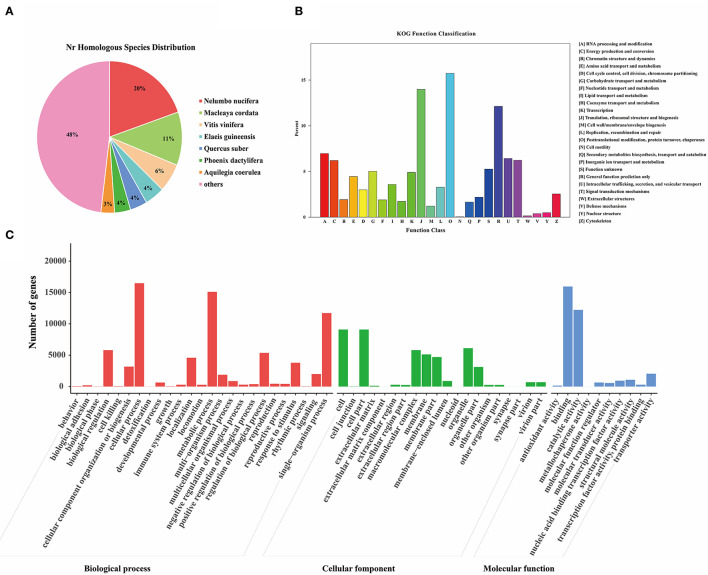
Functional annotations of the unigenes of the *M. microcarpa* transcriptome. **(A)** Species distribution of the NR results of *M. microcarpa* transcriptome. **(B)** KOG function annotation of unigenes. **(C)** GO function annotation of unigenes.

The unigene sequences of the *M. microcarpa* were compared with a general functional database (Supplementary Table S4). From the NR database, 36,352 (41%) annotated unigenes were obtained; 21,054 (23.74%), 14,044 (15.84%), 25,756 (29.05%), 29,166 (32.89%), 29,166 (32.89%), and 7,944 (8.96%) were obtained from the NT, KO, Swiss-Prot, Protein family (PFAM), Gene Ontology (GO), and microcarpa Ortholog Groups (KOG) databases, respectively. NR is the official protein sequence database of the NCBI. *M. microcarpa* transcripts were highly similar to *Nelumbo nucifera* (19.7%), *Macleaya cordata* (11.4%), and *Vitis vinifera* (6.1%) (Figure 3A). KOG is a database of orthologous gene families. A total of 7,944 unigenes of *M. microcarpa* were annotated to 25 KOG pathways (Figure 3B). A total of 1,250 unigenes were annotated to the post-translational modification, protein turnover, and chaperones, followed by 1,110, and 963 annotations related to gene expression, including translation, ribosomal structure and biogenesis, and general function prediction classification, respectively. GO is a gene function database. A total of 29,166 unigenes from *M. microcarpa* were annotated into 55 GO pathways (Figure 3C). The functions of unigenes in biological process classifications contained cellular process, metabolic process, and single-organism process. The cell, cell part, organelle, and macromolecular complex were the most abundant functions in terms of cellular component classifications. In the molecular function classification, binding, catalytic activity, and transporter activity were more abundant.

**Figure 4:**
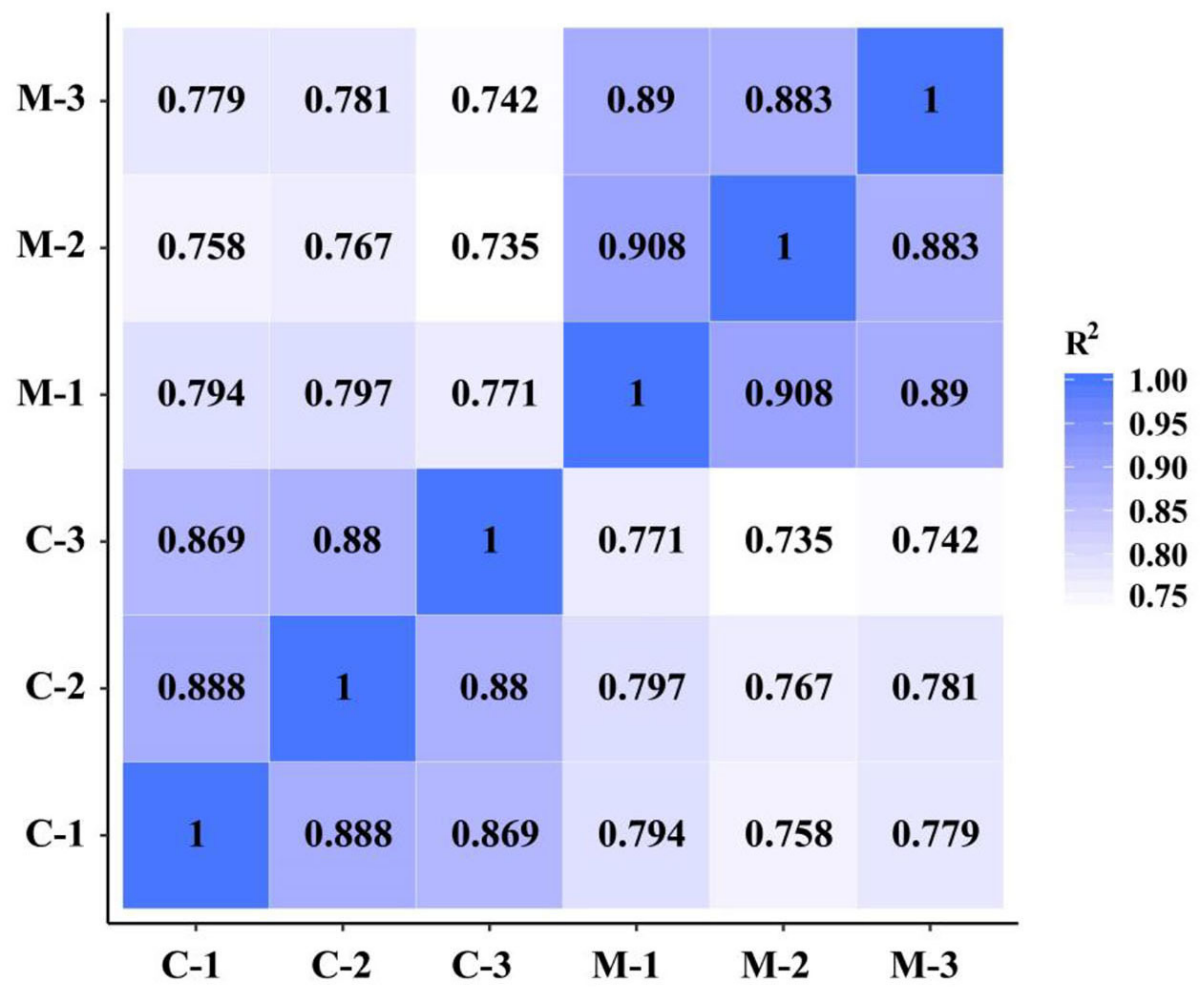
Pearson correlation between samples of the *M. microcarpa* transcriptome. The number in the color block represents the value of correlation coefficient. C, samples treated at 25°C; M, samples treated at −2.8°C.

To demonstrate the reliability and adequacy of sample selection, gene expression correlations between samples are very important. For biological replicate samples receiving the same treatment, Pearson correlation coefficients were always >0.8 (Figure 4), which indicated good reproducibility of the three biological replicates in each treatment.

### Identification and Functional Annotation of DEGs

**Figure 5:**
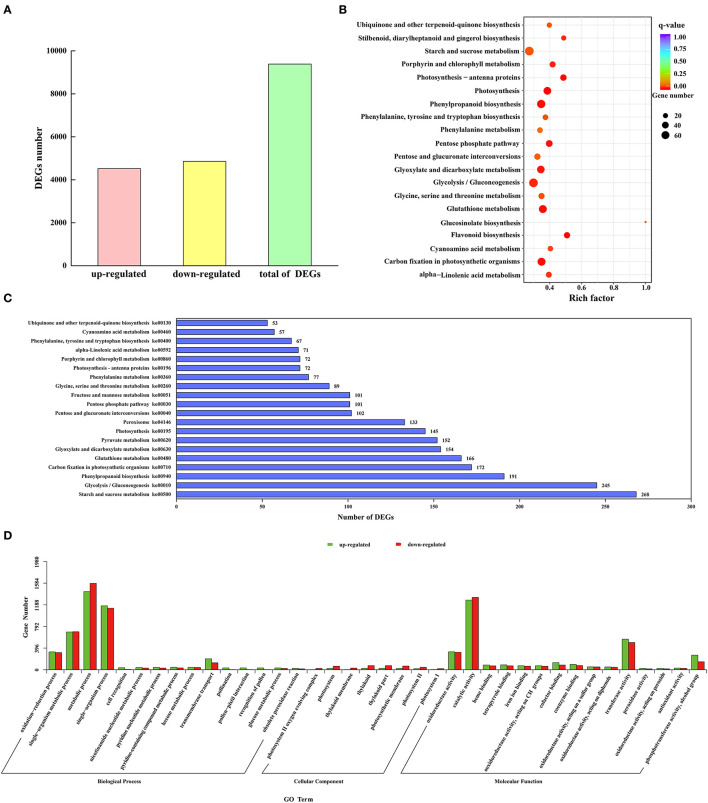
Identification and functional annotation of DEGs. **(A)** The number of upregulated and downregulated DEGs. **(B)** Q-value distribution of the top 20 enrichment pathways. **(C)** Enrichment of the top 20 KEGG pathways of all DEGs. **(D)** GO enrichment results of upregulated and downregulated DEGs.

To study the expression difference of unigenes in different treatments according to the expression level between the two groups of samples, the differential genes were divided into upregulated and downregulated genes. Through comparison, 9,385 DEGs were obtained, including 4,525 upregulated genes and 4,860 downregulated genes (Figure 5A).

A total of 38 functional annotations of the DEGs of M and C were obtained in the GO database (Figure 5D). The biological process contained 15 GO entries, mainly focusing on the metabolic process, single-organism process, and single-organism metabolic process, with 1,428, 1,169, and 691 upregulated genes, respectively, and 1,579, 1,125, and 695 downregulated genes, respectively. There were 8 GO entries in the cellular component, and the thylakoid and thylakoid parts were the main ones, with 28 upregulated genes and 75 downregulated genes, respectively. The molecular function had 15 GO entries, mainly distributed in catalytic activity and transferase activity, with 1,273 and 559 upregulated genes and 1,324 and 499 downregulated genes, respectively. Using significance enrichment analysis (FDR ≤ 0.05), DEGs were enriched in biological processes, cellular components, and molecular functions (Supplementary Table S5). The biological process was mainly enriched in metabolic process, single-organism process, single-organism metabolic process, and oxidation-reduction process. The cellular component was mainly enriched in the photosystem II oxygen-evolving complex. The molecular function was mainly enriched in catalytic activity, transferase activity, and oxidoreductase activity. To further analyze the DEGs in the *M. microcarpa*, functional annotation information statistics of the DEGs were produced. A total of 11, 277 DEGs were annotated on 120 KEGG pathways, of which 18 pathways were significantly enriched (*P* < 0.05). According to the top 20 q-value distribution (Figure 5B) and enrichment of the KEGG pathway statistics of the DEG number (Figure 5C), the flavonoid biosynthesis (ko00941) pathway was the most significantly enriched (*P* < 0.05). The number of DEGs involved in starch and sucrose metabolism (ko00500), glycolysis/gluconeogenesis (ko00010), and phenylpropanoid biosynthesis (ko00940) pathways were the highest, with 268, 245, and 191 DEGs, respectively. DEGs were also enriched in antioxidant enzyme-related pathways, such as phenylalanine metabolism (ko00360) and peroxisome (ko04146) pathways. These enable the mining of key genes for low-temperature tolerance in *M. microcarpa*.

### DEGs Related to Porphyrin and Chlorophyll Metabolism

**Figure 6:**
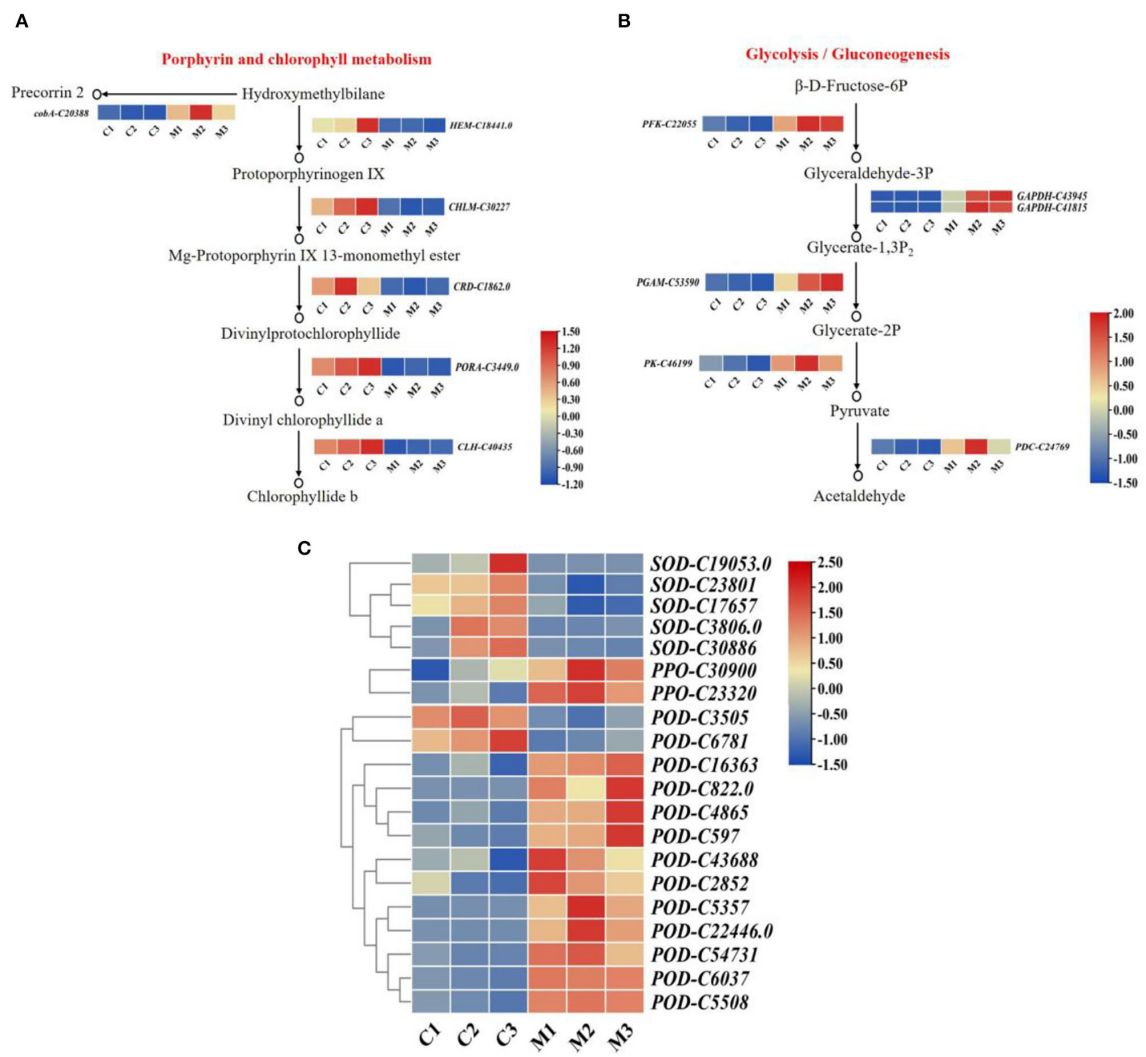
The expression patterns of DEGs. **(A)** The expression patterns of DEGs involved in porphyrin and chlorophyll metabolism pathway in *M. microcarpa*. **(B)** The expression patterns of DEGs involved in glycolysis/gluconeogenesis pathway in *M. microcarpa*. **(C)** Heat map depicting the expression patterns of antioxidant enzyme-related DEGs in *M. microcarpa*. Changes in expression level are indicated by a change in color, blue indicates a lower expression level, whereas red indicates a higher expression level. C, samples treated at 25°C; M, samples treated at −2.8°C.

To investigate the role of chlorophyll content in low temperatures. We determined that the highest content of chlorophyll was found in the control, followed by the low-temperature treatment (Figure 1C). Six DEGs were related to the porphyrin and chlorophyll metabolism pathway. The expression levels of six DEGs differed significantly between low-temperature treatment and control (Figure 6A). The expression levels of oxygen-dependent coproporphyrinogen-III oxidase (*HEM*), magnesium protoporphyrin IX methyltransferase (*CHLM*), magnesium-protoporphyrin IX monomethyl ester cyclase (*CRD*), chlorophyllase-2 (*CLH*), and protochlorophyllide reductase (*PORA*) were significantly lower in low-temperature treatment than in the control. Moreover, cluster analysis revealed that siroheme synthase (*cobA*) was significantly highly expressed in low-temperature treatment. Hence, the expression levels of *HEM, CHLM, CRD, CLH*, and *PORA* were downregulated by low-temperature treatment, and the synthesis of chlorophyll was reduced under low temperature during *M. microcarpa* leaves.

### DEGs Related to Glycolysis/Gluconeogenesis

At low temperatures, the content of soluble sugar increased compared with the control in the *M. microcarpa* leaves (Figure 1E). The KEGG annotations showed that six DEGs were related to the glycolysis/gluconeogenesis pathway. Heatmap analysis indicated that the expression levels of six DEGs differed significantly in low-temperature treatment than in the control (Figure 6B). Glyceraldehyde-3-phosphate dehydrogenase (*GAPDH*), ATP-dependent 6-phosphofructokinase (*PFK*), 2,3-bisphosphoglycerate-dependent phosphoglycerate mutase (*PGAM*), pyruvate decarboxylase (*PDC*), and pyruvate kinase (*PK*) were more highly expressed in low-temperature treatment. The expression levels of the DEGs were consistent with the trends of soluble sugar content, thus, these six DEGs might be important in promoting soluble sugar. Hence, low temperature could promote the expression level of key genes in the glycolysis/gluconeogenesis pathway in *M. microcarpa* leaves.

### DEGs Related to Antioxidant Enzyme

To identify DEGs involved in antioxidant enzymes, we determined the POD, SOD, and PPO activities in the *M. microcarpa* leaves. From the transcriptome, we found 20 unigenes encoding antioxidant enzymes. Among these 20 DEGs, the expression levels of 13 unigenes of *PPO-C30900, PPO-C23320, POD-C16363, POD-C822.0, POD-C4865, POD-C597, POD-C43688, POD-C2852, POD-C5357, POD-C22446.0, POD-C54731, POD-C6037*, and *POD-C5508* were significantly higher in the low-temperature treatment than in the control. Furthermore, the expression levels of *SOD-C19053.0, SOD-C23801, SOD-C17657, SOD-C3806, SOD-C30886, POD-C3505*, and *POD-C6781* were the lowest in the low-temperature treatment (Figure 6C). Hence, the expression levels of PPO and POD increased enzymatic activity in the *M. microcarpa* under low temperatures.

### Gene Expression Analysis of TFs

**Figure 7:**
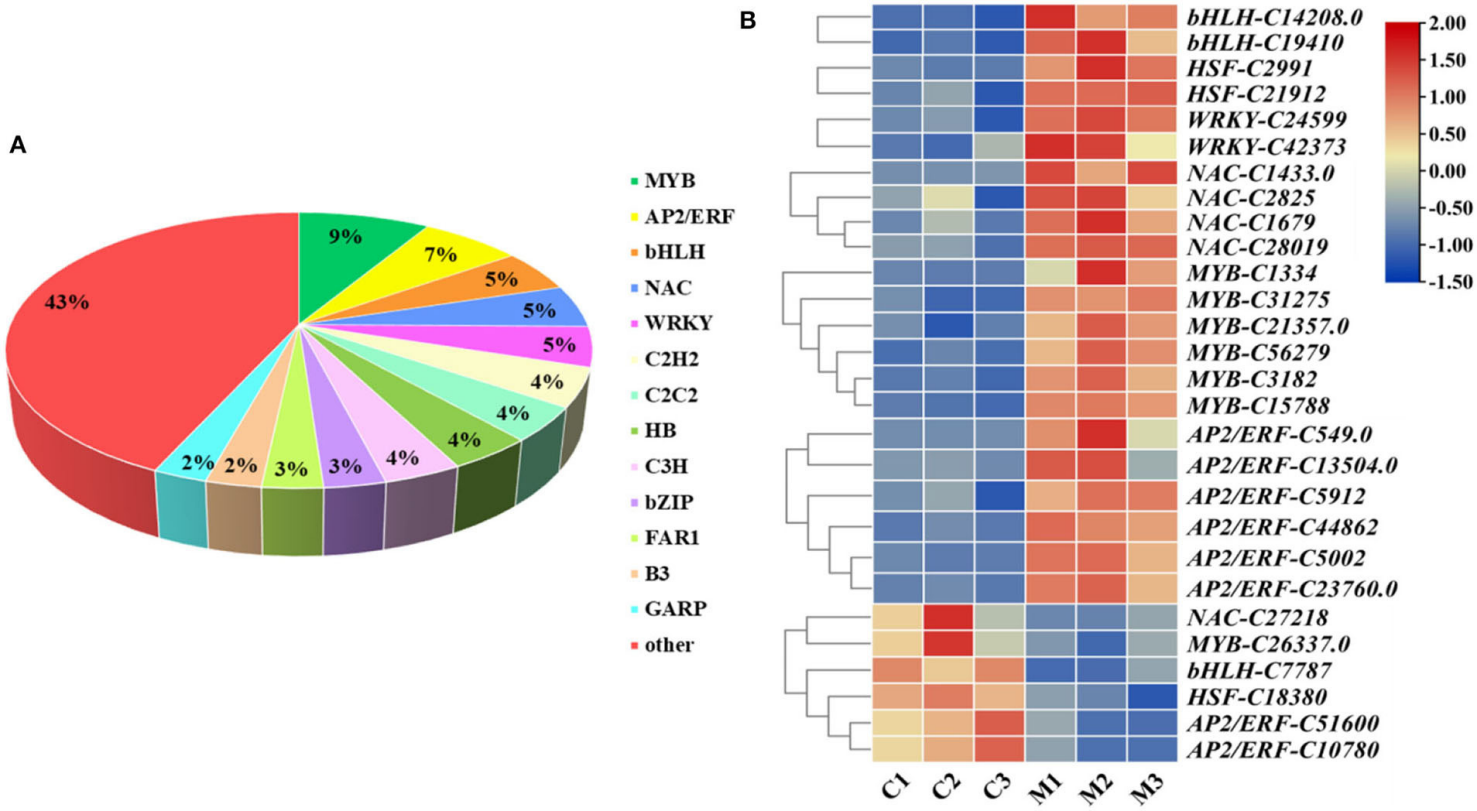
Analysis of differentially expressed TFs related to low-temperature stress. **(A)** Distribution of TF families in the transcriptome in *M. microcarpa* leaves. **(B)** Expression profiles of differentially expressed TFs. Changes in expression level are indicated by a change in color, blue indicates a lower expression level, whereas red indicates a higher expression level. C, samples treated at 25°C; M, samples treated at −2.8°C.

The gene expression network regulated by TFs plays a very important role in the regulation of plant abiotic stress. Overall, 1,745 TFs were annotated and classified into 62 families in *M. microcarpa*, 428 of which were differentially expressed (Figure 7A). To better understand the molecular mechanism of low-temperature treatment in *M. microcarpa*, we determined the differentially expressed TFs and obtained 28 TFs with significant differences from six TF families between the low-temperature treatment and CK (Figure 7B). Compared with the control, the results indicated that significantly upregulated TFs included two TFs belonging to the bHLH family (*bHLH-C14208.0, bHLH-C19410*), two TFs from the HSF family (*HSF-C2991, HSF-C21912*), two TFs from the WRKY family (*WRKY-C24599, WRKY-C42373*), four TFs from the NAC family (*NAC-C1433.0, NAC-C1679, NAC-C28019, NAC-C2825*), six TFs from the MYB family (*MYB-C1334, MYB-C56279, MYB-C31275, MYB-C3182, MYB-C15788, MYB-C21357*), and six TFs from the AP2/ERF family (*AP2/ERF-C549.0, AP2/ERF-C5002, AP2/ERF-C44862, AP2/ERF-C13504.0, AP2/ERF-C23760.0, AP2/ERF-C5912*). The significantly downregulated TFs included one NAC family (*NAC-C27218*), one MYB family (*MYB-C26337*), one bHLH family (*bHLH-C7787*), one HSF family (*HSF-C18380*), and two AP2/ERF families (*AP2/ERF-C51600, AP2/ERF-C10780*). These TFs may play a very important role in promoting the low-temperature treatment in *M. microcarpa*. Moreover, the low-temperature stress in *M. microcarpa* comprises a highly complex transcriptional network, and these results provide a basis for studying the role of TFs in low-temperature stress in *M. microcarpa*.

### Validation of DEGs by qRT-PCR

**Figure 8:**
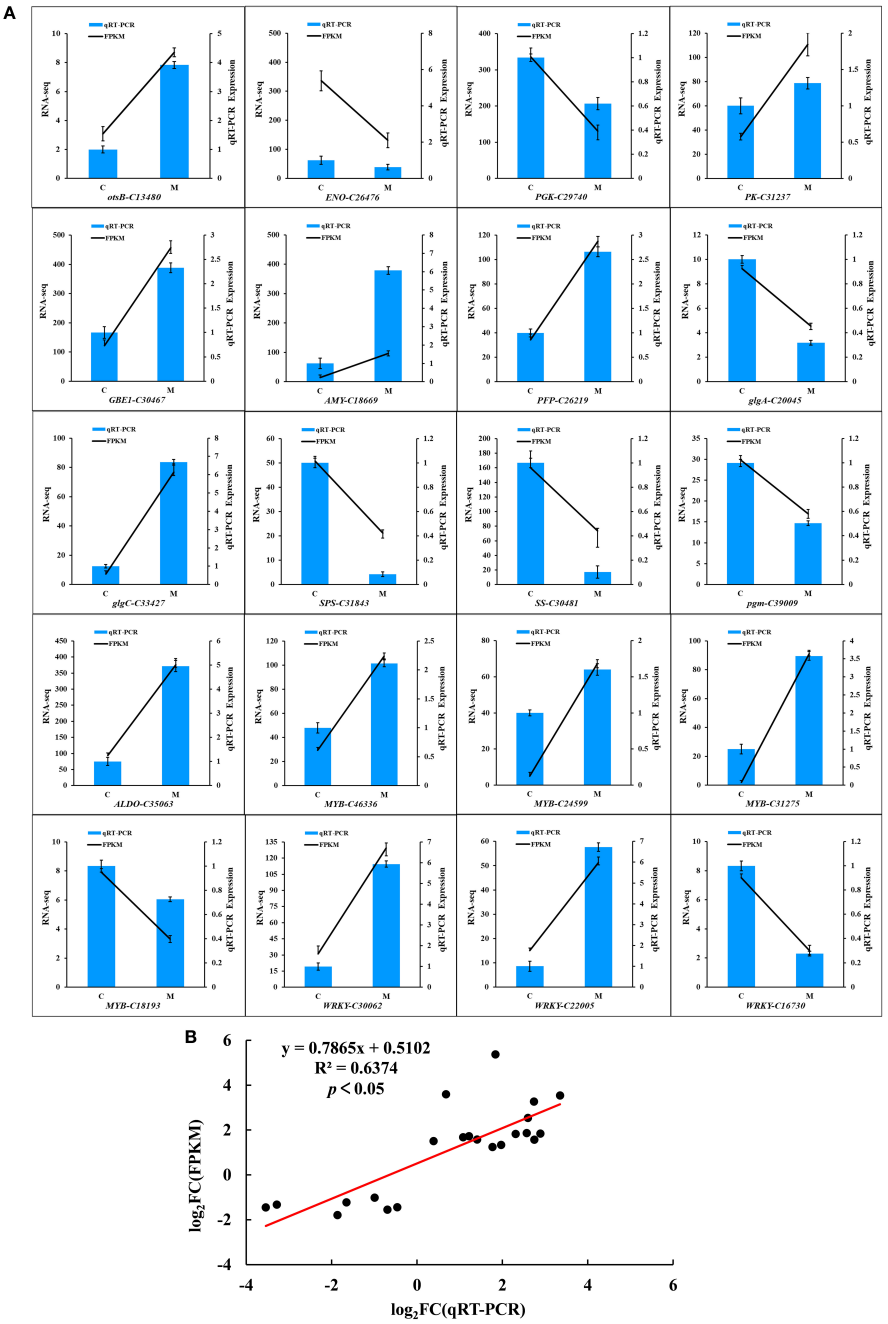
Quantitative Real-Time PCR validation of the 20 candidate genes in *M. microcarpa* transcriptome. **(A)** qRT-PCR validation of gene expression level in the transcriptome. The error bars represent the standard error of three biological replicates. Means with different letters in each tissue represent a significant difference at *p* ≤ 0.05. **(B)** Correlation analysis of the results between RNA-seq and qRT-PCR. Results were calculated using log_2_ fold variation measurements. The *R*^2^ value represents the correlation between the RNA-seq and qRT-PCR results.

To assess the accuracy of the RNA-seq data, 20 DEGs were randomly selected from the two groups of samples for qRT-PCR (Figure 8). The relative transcript levels of the low-temperature treatment and control were compared using qRT-PCR (Figure 8A). Among the 20 DEGs, qRT-PCR values of 11 genes (*otsB-C13480, PK-C31237, GBEI-C30467, AMY-C18669, PFP-C26219, glgc-C33427, ALDO-C35063, MYB-C46336, MYB-C24599, MYB-C31275*, and *WRKY-C30062*) were 3.9, 1.3, 2.3, 6, 2.6, 6.7, 4.9, 2.1, 1.6, 3.6, and 5.9 times higher, while 9 genes (*ENO-C26476, PGK-C29740, glgA-C20045, SPS-C31843, SS-C30481, pgm-C39009, MYB-C18193, WRKY-C22005*, and *WRKY-C16730*) were 7.4, 1.6, 3.2, 12.5, 10, 2, 1.4, 6.7, and 3.7 times lower in the treatment group than the control group in low temperature, respectively. As expected, a significantly positive correlation was observed between the qRT-PCR and transcriptome data in the M and C libraries (*R*^2^ = 0.6374, *p* < 0.05; Figure 8B). The transcriptome data were highly reproducible and reliable and could be used to further study the key genes related to tolerance to low temperatures in *M. microcarpa*.

## Discussion

### Physiological Changes in *M. microcarpa* Under Different Temperature Treatments

Conductivity is an important indicator of cell membrane permeability. The lower the cell tissue conductivity, the smaller the cell membrane permeability and the smaller the damage to cell membrane integrity (Yang et al., 2022). Measuring the electrolyte extravasation rate is one of the most effective methods for studying the cold tolerance of plants. This rate is combined with a logistic curve equation to derive the LT_50_, which can accurately reflect the low-temperature limit of plant tolerance (Yue et al., 2020); LT_50_ is widely used and applied to study the cold tolerance of plants (Zheng et al., 2015; Lu et al., 2017, 2019). In this study, the relative electrical conductivity of *M. microcarpa* leaves under different temperature treatments was measured, and the relative conductivity showed different trends. Under 25°C, the relative conductivity was the lowest. With the continuous decrease in temperature, the relative conductivity showed an “S” curve and an obvious upward trend. When the treatment temperature was −5°C, the relative conductivity was the highest, at more than 90%. This was consistent with the research conclusions of Armstrong et al. (2015) and Peixoto et al. (2015).

One of the reasons for the destruction of plants subjected to low-temperature stress is the lipid peroxidation of unsaturated fatty acids in the membrane induced by free radicals in cells (Wang et al., 2018). MDA is the final product of membrane lipid peroxidation, which represents an informative indicator of membrane damage (Cui et al., 2015). In this study, the malondialdehyde content in *M. microcarpa* leaves under different temperature treatments was measured. The malondialdehyde content showed an upward trend with a decrease in temperature. At 25°C, the content of MDA was the lowest. With a decrease in temperature, the content of MDA increased. When the temperature was reduced to −5°C, the MDA content was the highest, indicating that low-temperature stress had caused damage to the cells, leading to a large accumulation of MDA in *M. microcarpa*. This was consistent with the results of Zhang et al. (2016) on winter wheat, in which the MDA level increased in the overwintering period.

The chlorophyll content in plants directly affects the rate of photosynthesis and the use of light energy. It also affects the growth and development of the whole plant and has a great impact on normal metabolism in plants (Sharma et al., 2020). In this study, chlorophyll content showed a downward trend as the temperature decreased. At 25°C, the chlorophyll content was the highest. With a continuous decrease in temperature, the chlorophyll content gradually decreased, indicating that *M. microcarpa* has a relatively strong tolerance to low temperatures. Under low-temperature stress, the changes in leaf morphology might be related to the physiological dehydration caused by low temperature, while the changes in leaf color were related to the reduction of new chlorophyll synthesis and the destruction of the original chlorophyll under low temperatures.

Plants under environmental stress accumulate osmotic regulators, which help reduce cell osmotic potential, maintain cell expansion pressure, and improve the water absorption ability of cells (Farhangi-Abriz and Torabian, 2017). Soluble sugar and soluble protein are all osmotic regulatory substances in plant cytoplasm. Numerous studies have reported that cold stress induces the accumulation of osmotic substances in plants to improve cold resistance (Saladin et al., 2003; Wang et al., 2019). In this study, the variations in soluble sugars and soluble proteins showed different trends under different temperatures. At 12°C, the soluble protein content was the lowest. With the continuous decrease in temperature, the soluble protein content increased first and then decreased, possibly because *M. microcarpa* could adapt to environmental changes by regulating metabolic substances in plants at low temperatures. At 25°C, the soluble sugar content was the lowest, and with the decrease in temperature, the soluble sugar content showed a trend of increase–decrease–increase. At −5°C, the soluble sugar content was the highest, indicating that the soluble sugar content could be increased by inducing the activity of hydrolase at a low temperature.

Antioxidant enzymes are defensive enzymes when plants are subjected to stress conditions and play an important role in cell signaling and homeostasis (Wang et al., 2016). SOD, POD, and PPO are all important antioxidative enzymes that maintain the normal physiological activities of plants to a certain extent (Rahman et al., 2004; Kaur et al., 2009; Kusvuran et al., 2016). Many reports have confirmed that the capacity of antioxidative systems is a remarkable responsive mechanism for the cold tolerance of plants (Yang et al., 2008; Liu et al., 2010). In the present study, the POD, SOD, and PPO activities showed a consistent trend at different temperatures. With the decrease in temperature, POD, SOD, and PPO activities overall showed an increasing trend. There are reports that plants can resist cold damage by increasing the activity of antioxidant enzymes (Rivero et al., 2002). The POD, SOD, and PPO activities were the lowest at 12°C, and highest at −5°C. At low temperatures, plants initiate emergency mechanisms for the accumulation of free radicals to enhance their cold resistance, while low-temperature stress causes an increase in SOD, POD, and PPO activities.

### Role of Chlorophyll Metabolism in *M. microcarpa* Low-Temperature Stress

Transcriptome sequencing plays an important role in plant functional genomes, which can reflect differences in gene expression levels in different physiological states, different organs, or at different times. RNA-seq can identify many cold stress-responsive DEGs (Shen et al., 2014; da Maia et al., 2016; Yang et al., 2019).

Chlorophyll is an important pigment related to plant photosynthesis, as it plays a role in the plant's tolerance to cold stress. In *Brassica campestris*, comparative transcriptome analysis revealed that chlorophyll metabolism contributes to leaf color changes in response to cold (Yuan et al., 2021). The main significantly downregulated pathways were involved in porphyrin and chlorophyll metabolism related to cold resistance of *Lilium davidii* (Tian et al., 2020). In this study, six DEGs (*HEM, CHLM, CRD, CLH*, and *PORA* downregulated expression, and *cobA* upregulated expression) are involved in porphyrin and chlorophyll metabolism were identified from the RNA-seq data. The result showed that five key genes were downregulated under low-temperature stress, reducing chlorophyll synthesis in *M. microcarpa* leaves. The *cobA* was up-regulated expression, which played a defensive role under low-temperature stress, to improve the synthesis of chlorophyll and maintain the survival and growth of plants.

### Role of Sugar Metabolism in *M. microcarpa* Low-Temperature Stress

Sugars play an important role in plant cold tolerance and can function as signal molecules, participating in growth and various stress responses in plants (Yang et al., 2019; Saksena et al., 2020; Salvi et al., 2022). Many genes related to sugar synthesis have been identified in *Arabidopsis*, and these genes show differential expression during cold tolerance (Fowler and Thomashow, 2002). Several studies have shown that low temperatures induce starch degradation and that the genes encoding the major enzymes involved in this pathway are differentially regulated, promoting low-temperature responses (Li et al., 2011; Purdy et al., 2013). In the present study, six DEGs (two *GAPDHs, PFK, PGAM, PDC*, and *PK*) related to the glycolysis/gluconeogenesis pathway were identified from transcriptome analyses, which implied that these key genes are upregulated under low-temperature stress and promote the conversion of β-D-Fructose-6P to acetaldehyde (Figure 6B). Plants adapt to low-temperature stress by accelerating the oxidative decomposition of sugars. Sugar increased water retention and osmotic potential in cells and provided energy for the plant under adverse cold conditions (Yang et al., 2019).

### Role of Antioxidant Enzyme in *M. microcarpa* Low-Temperature Stress

Low-temperature stress promotes the accumulation of ROS, which are toxic substances that cause oxidative damage to plants (Sachdev et al., 2021). An enzymatic antioxidant system including superoxide dismutase, peroxidase, and polyphenol oxidase, provides an efficient and specific ROS scavenging system for plants. They play an essential role in protecting plants from oxidative damage by ROS (Mittler, 2002). In the current study, the expression levels of DEGs (two *PPOs* and 11 *PODs*) were significantly higher in the low-temperature treatment than in the control. However, the SOD genes were downregulated in response to low-temperature stress, a similar report was found in barley cultivars (Kayihan et al., 2012). The results indicated that although these POD, SOD, and PPO genes could display different expression roles under low-temperature stress, SOD genes played a critical and positive role in different plants' responses to various abiotic stresses. These genes perform unique functions under stressful conditions and play special functions under stress conditions, forming a complex antioxidant defense system *in vivo*.

### Candidate TFs Involved in *M. microcarpa* Low-Temperature Stress

TFs play an important role in plant response and resistance to stress (Gujjar et al., 2014). In this study, 1,745 TFs were annotated and classified into 62 families in *M. microcarpa*, 428 of which were differentially expressed. Among these TF families, bHLH, WRKY, NAC, MYB, and AP2/ERF accounted for a big proportion. The results were similar to those of the *Populus tomentosa* (Yang et al., 2019), *Betula platyphylla* (Yan et al., 2020), and *indica* rice (Pradhan et al., 2019) transcriptomes, in which WRKY, MYB, NAC, bHLH, HSF, and AP2/ERF TFs play regulatory effects on plant abiotic stress (Saha et al., 2015; Li et al., 2020).

Expression data from different plant species have indicated that members of the MYB family participate in plant responses to cold stress (Jiang et al., 2013; Tian et al., 2013). The ectopic expression of *Myb4* TF improves physiological and biochemical adaptation to cold stress and modifies metabolite accumulation in apples (Pasquali et al., 2008). *Arabidopsis thaliana* transgenic plants overexpressing *OsMYB3R-2* show increased tolerance to cold temperatures (Dai et al., 2007). The AP2/ERF is a large TF family in plants involved in plant developmental processes (Klay et al., 2018). The TF family includes DRE-binding proteins (DREB) and C-repeat binding factor (CBF). *AgDREB1* and *AgDREB2* contribute to the enhanced resistance to abiotic stress in transgenic *Arabidopsis* (Li et al., 2019b). In *Betula platyphylla, BpERF13* regulates physiological processes underlying cold tolerance (Yan et al., 2020). At present, many NAC TFs have been reported to be involved in plants' low-temperature stress. *MaNAC1* can enhance the cold tolerance in *Musa acuminata* (Shan et al., 2014). In the *Capsicum annuum*, the expression of *CaNAC2* seedlings increased at low temperatures (Guo et al., 2015). However, *MdNAC029* negatively regulated the cold tolerance of apples by inhibiting the expression of *MdCBF1* and *MdCBF4* (An et al., 2018). In this study, six *MYBs*, six *AP2/ERFs*, and four *NACs* were significantly upregulated, an MYB and NAC gene, and two *AP2/ERFs* were downregulated after low-temperature treatment. Therefore, when MYB, AP2/ERF, and NAC TFs participate in the regulation of low-temperature response, there may be positive and negative ways, among which the transcription factors with positive regulation account for the majority.

It also appears likely that WRKY, bHLH, and HSF TFs play an important role in *M. microcarpa* under low-temperature stress. There are many studies on how WRKY, bHLH, and HSF TFs respond strongly and rapidly to abiotic stress (Jiang et al., 2009; Chen et al., 2012; Ma et al., 2014). In *Verbena bonariensis, VbWRKY32*, as a positive regulator, upregulates the transcriptional level of cold response genes and improves survival ability under cold stress (Wang et al., 2020). In eggplant, *SmWRKY26* and *SmWRKY32* positively regulate the response to cold stress (Yang et al., 2020). Under low-temperature stress, *OsbHLH1* gene expression is induced in rice seedlings (Wang et al., 2003). *MdCIbHLH1* encodes a transcription factor that is important for the cold tolerance response in apples (Feng et al., 2012). Overexpression of *TaHSF3* in transgenic Arabidopsis enhances tolerance to extreme temperatures (Zhang et al., 2013). In this study, two of the *WRKYs, bHLHs*, and *HSFs* were significantly upregulated after low-temperature treatment, thereby indicating that these TFs might play some positive regulatory roles in *M. microcarpa*.

In summary, combined physiological indexes and transcriptome sequencing showed that the low-temperature tolerance of *M. microcarpa* may occur through the synthesis of cold-inducible TFs initiating a series of cellular pathways, for example, increasing soluble sugar and other osmotic regulatory substances to resist external stress and producing SOD, POD, and PPO antioxidant enzymes to eliminate excessive free radicals generated in cells due to low-temperature stress. In this process, many enzyme genes are expressed, initiating a series of cell activities against external environmental stress.

## Conclusions

**Figure 9:**
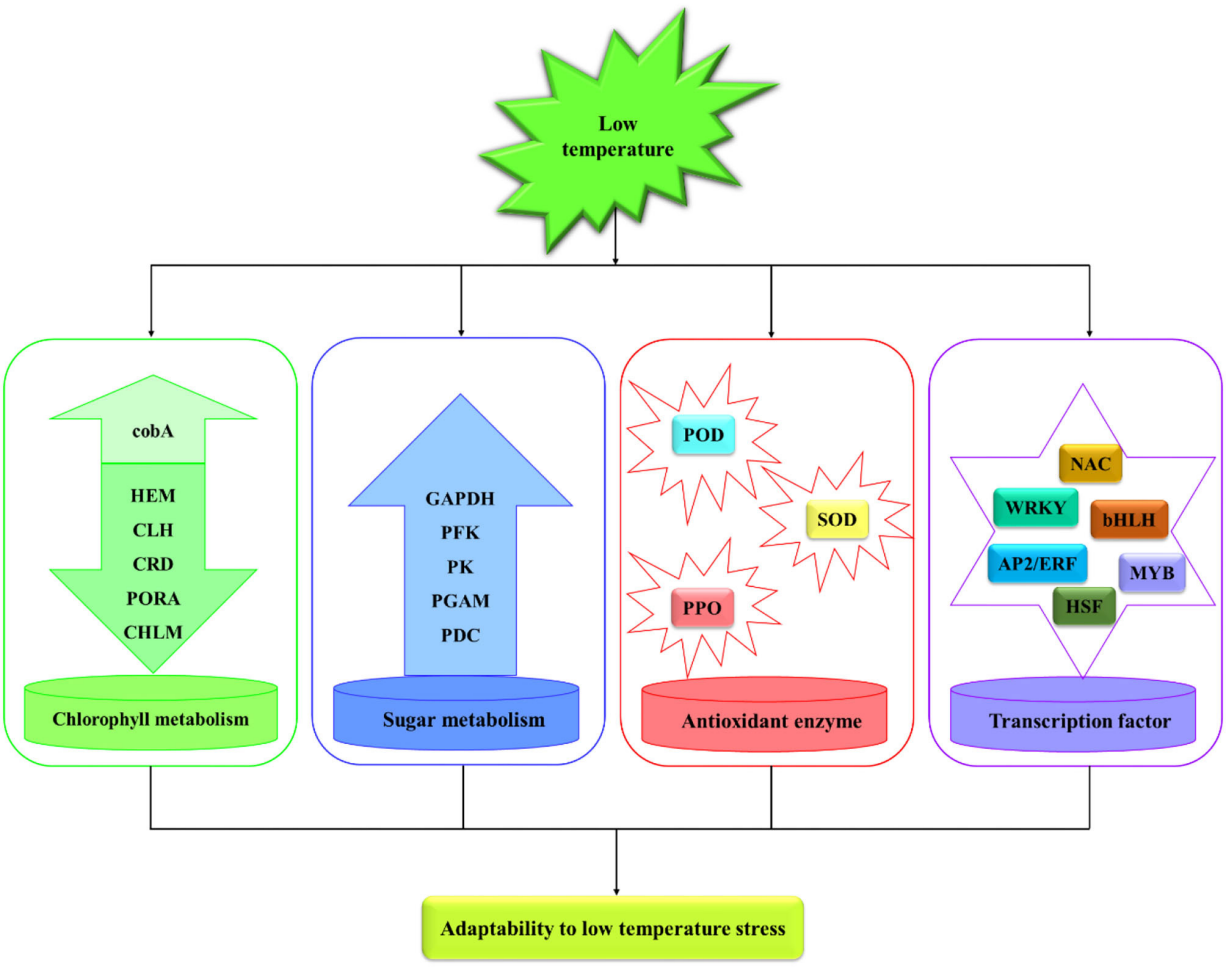
Regulation network model in *M. microcarpa* leaves under low-temperature stress.

In this study, annual *M. microcarpa* plants were treated at 25, 12, 5, 0, and −5°C. The relative electric conductivity of *M. microcarpa* leaves increased with decreasing temperature, and the LT_50_ was −2.8°C. The total chlorophyll content decreased, and the contents of MDA and soluble sugar and the activities of POD, SOD, and PPO increased and were used as the indexes of cold tolerance in *M. microcarpa*. POD, SOD, and PPO activities and soluble sugar content play an important role in the scavenging of active oxygen substances in *M. microcarpa*. Transcriptome data of *M. microcarpa* leaves revealed the molecular regulation of low-temperature tolerance. There were 9,385 DEGs involved in low-temperature stress in the *M. microcarpa* transcriptome data. Some DEGs were related to chlorophyll, sugar, and antioxidant enzyme, and some important TFs were involved in *M. microcarpa* under low-temperature stress (Figure 9). Differential expression analysis indicated that low-temperature stress downregulated key genes involved in the porphyrin and chlorophyll metabolism pathway, thus, reducing chlorophyll synthesis in *M. microcarpa* leaves. The expression levels of key genes also related to sugar metabolism and antioxidant enzyme were influenced by low-temperature treatment. In addition, some TFs, such as MYB, AP2/ERF, NAC, WRKY, bHLH, and HSF genes played an important role under low-temperature stress in *M. microcarpa* leaves. The transcriptome data can provide a basis for further analysis of the molecular mechanisms of low-temperature stress in *M. microcarpa*.

## Data Availability Statement

The datasets presented in this study have been deposited in the Genome Sequence Archive (Wang et al., 2017) in National Genomics Data Center (CNCB-NGDC Members and Partners, 2022), China National Center for Bioinformation/Beijing Institute of Genomics, Chinese Academy of Sciences, under accession number CRA006331 that are publicly accessible at https://ngdc.cncb.ac.cn/.

## Author Contributions

YF and XH designed the research. XH wrote the manuscript. LH, TY, and YH collected the experimental materials. XH, FL, YL, and YX completed the experiment. DH and YY helped with the experiment. YF revised the manuscript. XH analyzed all the materials in this research. All authors contributed to the article and approved the submitted version.

## Funding

This work was supported by the Science and Technology Research Project of the Education Department in Hubei Province of P.R. China (Q20181314) and the Natural Science Foundation Project in Hubei Province of P.R. China (2017CFB390).

## Conflict of Interest

The authors declare that the research was conducted in the absence of any commercial or financial relationships that could be construed as a potential conflict of interest.

## Publisher's Note

All claims expressed in this article are solely those of the authors and do not necessarily represent those of their affiliated organizations, or those of the publisher, the editors and the reviewers. Any product that may be evaluated in this article, or claim that may be made by its manufacturer, is not guaranteed or endorsed by the publisher.
