# Supplementary material for: Comparative Transcriptome Analysis Revealing the Potential Mechanism of Low-Temperature Stress in Machilus microcarpa
Source: Front Plant Sci. 2022 Jul 19;13:900870. doi: 10.3389/fpls.2022.900870 (PMC9348548; doi:10.3389/fpls.2022.900870)
Supplement: Supplementary file 1 [file Table_1.DOCX]

**Table S1. Primers used in qRT-PCR**

| **Gene ID** | **Sense primer sequence（5' to 3'）** | **Antisense primer sequence（5' to 3'）** |
| --- | --- | --- |
| *SPS-C31843* | CCTATGGTTTACCCGTTGTTG | GATGTTCTTGAGGCCAGTTTT |
| *AMY-C18669* | TCTGGCTTCCTCCACCTTCAC | CCCTCTTCCATCCTTCTTTTC |
| *OtsB-C13480* | GGGACACTTTCACCAATCGT | ACTTCCTTGCCCTGCTCATC |
| *PFP-C26219* | CATTACAGAGCATCCCCCAT | TTCGTCCGTCCTAGCAAGTC |
| *PK-C31237* | GCCTTACAGAGCCACAAGCA | AACGCCAAGAATCTCACCAG |
| *Pgm-C39009* | TGACAGTGCCCACAAAACCA | CCTCCTAAAGCCAGCAAACC |
| *GBE1-C30467* | AAGTACTATCACCAGCTCGCAC | AAGTCAACATCTTCCTCCTCAA |
| *SS-C30481* | AGTTCCTCAACCGCCACCTT | GTATCGCCCCAGCCTTTCTC |
| *GlgA-C20045* | TGTTCCATGTGGTGGTGTTT | ACTATGCGGTCGGCAGTTTT |
| *GlgC-C33427* | ATCTGGGGCAAATTGGTAATG | GGTGAGCGGAAAAAGGTGAGT |
| *ALDO-C35063* | GCTACAAGGCACTCAACGACC | GCCCACCTGACAAGAACACAA |
| *GpmI-C27257* | TAGAAGCGACAGTTGTGGGTTG | CTGGTTGTAGGGTATGCGAGGT |
| *PGK-C29740* | CAAATCAGCCCTCACGAACAC | CAACACCCACCTCCCTCTCTC |
| *ENO-C26476* | ACTGATTACCCGATTGTTTCC | CTTTTCCTTGATTGCTTTCCC |
| *MYB-C46336* | TCAGCAAGAGGCTCAAAACC | CGATGGACCACTTCCAAAAT |
| *MYB-C24599* | ACGACACCCATACGACGACAG | TTAGGAACTCCACCCAAACCC |
| *MYB-C31275* | AACTAGCGACCTGCAAACGG | CCAAGAGGAACACCACCACC |
| *MYB-C18193* | GATGTGGGAAGAGTTGTCGC | TTGAAGAGGTTTGTGGTGGC |
| *WRKY-C30062* | ATTACACCTCCCTCTCTACCCA | GCAGTTCCTCTCTTCCTTCTTG |
| *WRKY-C22005* | CCGCCTCTGTCATCGTCTTC | TTCTGCCCGTATTTCCTCCA |
| *WRKY-C16730* | AGGCAAAGCAGGTGGTTTGT | TGGATGATTGTGTTCGGAGG |
| *Ef-1a-*C11786.0 | GATACTCTTCCTCAGCGGTCA | CAGTCTTCCCCTTTACACGTT |
