# Supplementary material for: Comparative Transcriptome Analysis Revealing the Potential Mechanism of Low-Temperature Stress in Machilus microcarpa
Source: Front Plant Sci. 2022 Jul 19;13:900870. doi: 10.3389/fpls.2022.900870 (PMC9348548; doi:10.3389/fpls.2022.900870)
Supplement: Supplementary file 2 [file Table_2.DOCX]

**Table S2. Correlation analysis of physiological indexes under different temperature treatments**

|  | **CHL** | **SP** | **POD** | **SOD** | **PPO** | **MDA** | **REC** | **SS** |
| --- | --- | --- | --- | --- | --- | --- | --- | --- |
| **CHL** | 1 |  |  |  |  |  |  |  |
| **SP** | 0.036 | 1 |  |  |  |  |  |  |
| **POD** | -0.402 | 0.596* | 1 |  |  |  |  |  |
| **SOD** | -0.14 | 0.583* | 0.524* | 1 |  |  |  |  |
| **PPO** | -0.684** | 0.595* | 0.758** | 0.686** | 1 |  |  |  |
| **MDA** | 0.156 | 0.014 | 0.435 | 0.034 | 0.045 | 1 |  |  |
| **REC** | 0.497 | 0.249 | -0.226 | 0.260 | -0.126 | -0.366 | 1 |  |
| **SS** | 0.786** | 0.003 | -0.589 | -0.424 | -0.692** | -0.115 | 0.305 | 1 |

**Indicates that the correlation is significant (*P* < 0.01), *Indicates that the correlation is significant (*P* < 0.05), CHL: chlorophyll contents; SP: soluble protein; POD: peroxidase; SOD: superoxide dismutase; PPO: polyphenol oxidase; MDA: malondialdehyde; REC: relative electric conductivity; SS: soluble sugar.
