# Supplementary material for: Comparative Transcriptome Analysis Revealing the Potential Mechanism of Low-Temperature Stress in Machilus microcarpa
Source: Front Plant Sci. 2022 Jul 19;13:900870. doi: 10.3389/fpls.2022.900870 (PMC9348548; doi:10.3389/fpls.2022.900870)
Supplement: Supplementary file 3 [file Table_3.DOCX]

**Table S3. Quality inspection of sample sequencing data**

| **Sample** | **Raw Reads** | **Clean Reads** | **Clean Bases** | **Q30 (%)** | **GC（%）** |
| --- | --- | --- | --- | --- | --- |
| 25℃-C1 | 69891774 | 68023484 | 10.2G | 93.69 | 46.61 |
| 25℃-C2 | 67975980 | 66308158 | 9.95G | 93.59 | 47.16 |
| 25℃-C3 | 66484330 | 64284498 | 9.64G | 93.43 | 46.91 |
| -2.8℃-M1 | 67733138 | 65811142 | 9.87G | 93.3 | 46.15 |
| -2.8℃-M2 | 70857010 | 68883700 | 10.33G | 93.1 | 46.18 |
| -2.8℃-M3 | 71805294 | 70087954 | 10.51G | 93.29 | 46.14 |
| Average | 69124587.67 | 67233156 | 10.08 G | 93.4 | 46.525 |
