# Supplementary material for: Comparative Transcriptome Analysis Revealing the Potential Mechanism of Low-Temperature Stress in Machilus microcarpa
Source: Front Plant Sci. 2022 Jul 19;13:900870. doi: 10.3389/fpls.2022.900870 (PMC9348548; doi:10.3389/fpls.2022.900870)
Supplement: Supplementary file 4 [file Table_4.DOCX]

**Table S4. Functional annotations of unigenes in the NR, NT, KO, Swiss-Prot, PFAM, GO and KOG databases**

| **Annotated in database** | **Number of Unigenes** | **Percentage/%** |
| --- | --- | --- |
| NR | 36352 | 41% |
| NT | 21054 | 23.74% |
| KO | 14044 | 15.84% |
| Swiss-Prot | 25756 | 29.05% |
| PFAM | 29166 | 32.89% |
| GO | 29166 | 32.89% |
| KOG | 7944 | 8.96% |
