# Supplementary material for: Comparative Transcriptome Analysis Revealing the Potential Mechanism of Low-Temperature Stress in Machilus microcarpa
Source: Front Plant Sci. 2022 Jul 19;13:900870. doi: 10.3389/fpls.2022.900870 (PMC9348548; doi:10.3389/fpls.2022.900870)
Supplement: Supplementary file 5 [file Table_5.DOCX]

**TABLE S5. GO enrichment analysis of DEGs**

| **GO ID** | **Term type** | **Description** | **DEGs** |
| --- | --- | --- | --- |
| GO:0072524 | Biological process | pyridine-containing compound metabolic process | 78 |
| GO:0055114 | Biological process | oxidation-reduction process | 640 |
| GO:0046496 | Biological process | nicotinamide nucleotide metabolic process | 72 |
| GO:0044710 | Biological process | single-organism metabolic process | 1386 |
| GO:0044699 | Biological process | single-organism process | 2294 |
| GO:0019362 | Biological process | pyridine nucleotide metabolic process | 73 |
| GO:0008152 | Biological process | metabolic process | 3007 |
| GO:0008037 | Biological process | cell recognition | 42 |
| GO:0009654 | Cellular component | photosystem II oxygen evolving complex | 26 |
| GO:0050662 | Molecular function | coenzyme binding | 176 |
| GO:0048037 | Molecular function | cofactor binding | 217 |
| GO:0046906 | Molecular function | tetrapyrrole binding | 160 |
| GO:0020037 | Molecular function | heme binding | 157 |
| GO:0016740 | Molecular function | transferase activity | 1058 |
| GO:0016705 | Molecular function | oxidoreductase activity, acting on paired donors, with incorporation or reduction of molecular oxygen | 137 |
| GO:0016616 | Molecular function | oxidoreductase activity, acting on the CH-OH group of donors, NAD or NADP as acceptor | 95 |
| GO:0016614 | Molecular function | oxidoreductase activity, acting on CH-OH group of donors | 106 |
| GO:0016491 | Molecular function | oxidoreductase activity | 646 |
| GO:0005506 | Molecular function | iron ion binding | 138 |
| GO:0003824 | Molecular function | catalytic activity | 2597 |
